# Predicting substantive biomedical citations without full text

**DOI:** 10.1073/pnas.2213697120

**Published:** 2023-07-18

**Authors:** Travis A. Hoppe, Salsabil Arabi, B. Ian Hutchins

**Affiliations:** ^a^Office of the Director, National Center for Health Statistics, Centers for Disease Control and Prevention, Hyattsville, MD 20782; ^b^Information School, School of Computer, Data, and Information Sciences, College of Letters and Science, University of Wisconsin-Madison, Madison, WI 53706

**Keywords:** science policy, machine learning, citation analysis, artificial intelligence, bench to bedside translation

## Abstract

Insights from biomedical citation networks can be used to identify promising avenues for accelerating research and its downstream bench-to-bedside translation. Citation analysis generally assumes that each citation documents substantive knowledge transfer that informed the conception, design, or execution of the main experiments. Citations may exist for other reasons. In this paper, we take advantage of late-stage citations added during peer review because these are less likely to represent substantive knowledge flow. Using a large, comprehensive feature set of open access data, we train a predictive model to identify late-stage citations. The model relies only on the title, abstract, and citations to previous articles but not the full-text or future citations patterns, making it suitable for publications as soon as they are released, or those behind a paywall (the vast majority). We find that high prediction scores identify late-stage citations that were likely added during the peer review process as well as those more likely to be rhetorical, such as journal self-citations added during review. Our model conversely gives low prediction scores to early-stage citations and citation classes that are known to represent substantive knowledge transfer. Using this model, we find that US federally funded biomedical research publications represent 30% of the predicted early-stage (and more likely to be substantive) knowledge transfer from basic studies to clinical research, even though these comprise only 10% of the literature. This is a threefold overrepresentation in this important type of knowledge flow.

## Significance

Citation networks document knowledge flow across the biomedical literature, and insights from these networks are increasingly used to form science policy decisions. However, many citations are known to be not substantively related to critical early stages of the citing study. This adds noise to the insights derived from these networks. Here, we train a machine learning model that generates prediction scores associated with substantive citations. We use this model to show that government funding is linked to a disproportionate amount of knowledge transfer from basic to clinical research that is likely to be substantive in nature. This result raises the possibility that federal funding for biomedical research may be a straightforward lever for translating basic research knowledge into clinical discoveries.

United States (US) federal science funders, research institutions, and investigators seek to advance the frontiers of knowledge to stimulate innovation, improve human health, and maintain an edge in the global knowledge economy (1). Recently, the generation of large open datasets of grants, publications, clinical trials, and patents have facilitated the generation of linked knowledge networks that relate resources to knowledge creation and eventual flow into these applied outcomes (2–5). Insights from these networks can be used to identify promising avenues for accelerating research and its downstream applied impacts (6). However, despite the power of analyzing large-scale knowledge flow to inform decision-making, these efforts are complicated by one simple fact: Many, if not most, of citations in the scientific literature represent transfer of information that did not directly influence the conduct of a research study (see below). This makes it more challenging than would otherwise be the case to identify the subset of citations in the literature that really drove subsequent advancements in discovery.

Some citations document the provenance of information that was used to inform the inception, design, or execution of the main experiments of the new study. Such citations are often referred to as “substantive” citations (7). These may not be the only kind of “important” citation. But they are particularly interesting because the prior work was used in such a way that the experiments and results would have been different (or not even performed) without the prior knowledge being cited. In this case, an author’s reason for citing would be to acknowledge the intellectual impact of these studies on their current work (8). However, other citations are exclusively rhetorical in nature (9). A reason for an author to include such a citation might be to place new results in the context of other studies in the field or to showcase the novelty of the new discovery (10). In some cases, authors might hope to better persuade reviewers and editors to accept the paper for publication by including certain citations (11). These references are important to scientific communication, but lack a similar substantive nature. A small proportion of references track ideas that the authors consider, but then reject. Such “negative” citations are estimated to be < 10% of the references in the literature (12–14), a figure that may vary in degree by field. Negative citations are still a marker of scientific influence, since they are thought to accelerate the self-correcting nature of science (12). Finally, some references are strategic in nature, designed to game citation metrics. Often, self-citations are viewed through this lens (15), although this interpretation is debatable (16, 17). The detection and omission of rhetorical references could be an avenue for improving signal to noise in tracing the provenance of important scientific ideas.

Given the interest in distinguishing between substantive vs. rhetorical references, we asked whether it is possible to identify subsets of the citation literature that are unequally associated with substantive vs. rhetorical references. We then sought to use these as training data to build a scalable model that might indirectly identify substantive vs. rhetorical citations.

Prior work has focused on identifying the type of discourse surrounding the reference in text (known as the “citation context”), as a proxy for author intent. Such models have made significant advancements in predictive accuracy in recent years (9, 18–22). However, approaches using citation contexts face a major limitation: They require full text as an input, but most articles are still not open access. This limitation means that even if hypothetical models were perfect, they could only classify a small minority of citations. In addition, indexing of full text does not always improve information retrieval systems but can instead introduce unwanted noise (23). In biomedicine, the PubMed Central repository of public access articles captured 7 of the 32 million biomedical research articles in PubMed in 2021. This means that approximately 80% of substantive citations in biomedical research would be overlooked even with a perfectly accurate model.

Large-scale identification of the subset of citations that are likely to be substantive in nature without full text is not a solved problem (8, 24–26). However, researchers have identified subsets of citations that may be more- or less-concentrated with substantive vs. rhetorical citations. Authors’ reasons for including a citation may vary based on the stage at which a citation is added to a manuscript draft. Many references that influenced the study motivation or that provided key methodologies would out of necessity be collected at an early stage and included in initial drafts. Conversely, strategic rhetorical citations to the journal which is reviewing a late-stage manuscript are unlikely to be added until a very late stage, perhaps at submission or review. These conceptual associations raise the possibility of linking the stage at which a reference was added to its likelihood of being substantive vs. rhetorical.

Survey data asking researchers about the importance of the references that they themselves added to their publications show that those added late in the writing process are far less likely to be important for the inception, design, or execution of the main body of experiments (7). Self-reported scores on a 5-point Likert scale show a 50% higher rating in a reference’s influence on an author’s study if that reference was added at an early stage rather than during peer review. One study found that only approximately one out of twenty references was essential (8), although it should be noted that in their survey, the authors specifically requested 1 to 4 essential references per paper (which would amount to on the order of 5%). Estimates vary based on field of research, but rhetorical references account for 40 to 63% (7, 21, 27).

**Fig. 1.**
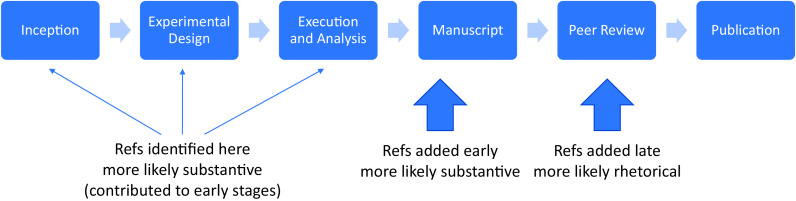
Proposed mental model of the testable relationship between citations added at early vs. late stages and their potential association with being substantive or rhetorical in nature.

Here, we sought to overcome this major limitation of coverage. We asked whether a combination of public-domain title/abstract text, article metadata, and the public domain citation graph (2, 28), contains any predictive information that could differentiate between classes of citations that are associated with being substantive vs. rhetorical. Such information is not nearly as rich as those available in citation contexts, which reveal not only the text surrounding a citation and its sentiment, but also the number of times a reference was cited within a paper and in which sections of the manuscript these are found. For this reason, this design approach is likely to explicitly trade-off high accuracy for a massive improvement in coverage that is a result of relying exclusively on comprehensive public-domain information. Early-stage references (i.e., those found in the original preprint) and late-stage references (i.e., those added during peer review) are thought to be associated with substantive vs. rhetorical characteristics. We take an indirect approach that has only recently become available at scale in biomedical research: biomedical preprints indexed in bioRxiv. Here, we describe a model that is trained to identify such early- vs. late-stage references. We test how early-stage vs. late-stage prediction scores from our model associate with known classes of substantive vs. rhetorical citations (Fig. 1). We find that this model exploits the latent relationship between early- and late-stage references and their rhetorical or substantive nature, but because of the indirect nature of this modeling approach, does so with lower accuracy than might be expected if free full text could be leveraged. We then apply our model to references from clinical articles. We studied the resultant prediction scores to gain insights into the efficacy of government funding (defined, for US biomedical research in this paper, as NIH funding for research articles indexed in PubMed) in stimulating early-stage (and therefore more likely to be substantive) flow of information from basic to clinical research.

## Results

### Identification of Late-Stage Citations.

To index citations that were present in biomedical preprints, we downloaded the full set of biomedical preprints from bioRxiv linked to peer-reviewed versions, identified using the Europe PubMed Central application programming interface. Citations were resolved with the Hydra citation resolution service (4). We then cross-referenced these preprint citations with those appearing in the peer-reviewed, published versions of the same paper using the NIH Open Citation Collection (2). In general, 11.6% of citations that were present in the peer reviewed version of the paper were added during review.

Most publications do not have preprints with which to compare reference lists. However, it may be possible to identify patterns in the citation network and metadata of a paper and its references that represent citations that were added at a late stage. This problem is challenging because citation dynamics are noisy and therefore difficult to analyze. However, citation dynamics do follow certain mathematical principles (29, 30). By combining measures of article content with measurements of citation dynamics, it is possible to detect enough statistical regularity to predict, years in advance, outcomes like knowledge transfer from basic to clinical research (3). Therefore, it stands to reason that there may be enough statistical regularity in an article’s metadata to differentiate between early- and late-stage references using machine learning, and then test whether these are associated with known classes of substantive vs. rhetorical citations.

### Feature space.

In order to generate a feature space in which a machine learning classifier could detect patterns in the public data that are associated with a reference being added during peer review vs. appearing in the original preprint, we considered information in three categories. The first category is metadata about the citing and referenced articles themselves. These included publication years of each paper, the degree of Human, Animal, and Molecular/Cellular Biology focus for each paper (3, 31), whether the citing and referenced paper appeared in the same journal, and whether the citing and referenced papers are primary research articles as defined in the NIH iCite tool (32).

The second category of information we included was information about the citation network and relationship between the citing and referenced articles. To encode this information, we generated measures that reflect size of the network, its growth rate, and particular types of citation relationship. First, we included information about the citing and referenced articles’ Relative Citation Ratio (RCR), which is a field- and time-normalized metric of an article’s citation rate (33, 34). Since its dissemination, RCR has been used by NIH in its portfolio analysis supporting science policymaking, strategic planning, and as evidence of good stewardship of taxpayer funds in U.S. Congressional Appropriations hearings supporting increases to the NIH budget (35–38). We also included information about ranking of these RCRs relative to the NIH-funded publication portfolio (here termed the RCR Percentile, but also referred to as NIH Percentile).

Previous work showed that highly cited papers are more likely to be the recipients of substantive citations as identified by authors (7). We make a confirmatory observation; the RCR percentile of papers that were cited in the original preprint was 2.4% higher than for papers whose reference was added during review (*P* < 0.001, Wilcoxon Rank Sum test). This is not because older papers have had more time to accrue citations. The relative age of citations added in peer review was lower (6 y gap) than for citations originally found in the preprint version (7 y gap, *P* < 0.001, Wilcoxon Rank Sum test). In addition, RCR percentile time-normalizes its measure of scientific influence, so this comparison would not be affected by differences in publication age. Instead, references to papers added in peer review appear to have notably lower citation influence when adjusting for field and time.

**Fig. 2.**
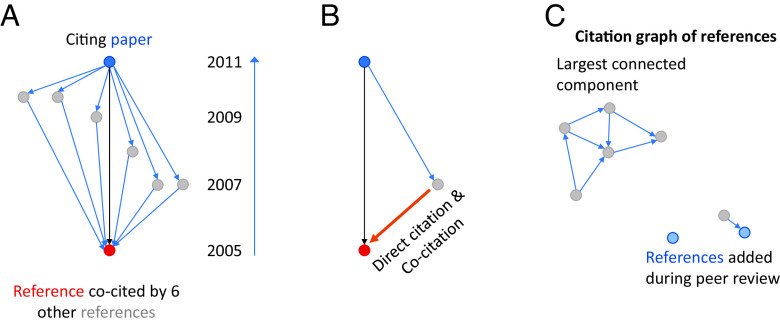
Local citation network features that may hold predictive power. (*A*) Illustration of references (gray) of a citing paper (blue) that all cocited the target referenced paper (red) found in the same citing paper’s reference list. In this case, six other papers from this reference list (gray) cocited the referenced article (red), so the count is six. (*B*) Illustration of a direct citation that is also a cocitation. The blue paper cites both the other citing (gray) and referenced (red) articles, making this orange direct citation link also a cocitation. (*C*) The local citation network of the papers found in the reference list. Many referenced papers appear in the largest connected component, but in this illustration the two papers (blue) that were added to the reference list during peer review are not part of this component.

We considered second-order local information about the citation relationship (that is, two steps away from the target publication on a citation) and included three additional measures of the citation network. First, we identified the number of other articles in the citing paper’s reference list that also cited the referenced paper (Fig. 2*A*). This could be interpreted as a measure of the authoritativeness of the referenced paper, since these other related works deemed it important enough to cite. Next, we identified whether a paper published after the two papers in question has subsequently cited both the citing and referenced papers (i.e., indicating the citation in question is both a direct citation and a cocitation). A direct and cocitation relationship (Fig. 2*B*) could be an indication that the two papers have been built upon and utilized together in a later scientific project. Third, we considered the local citation network of the full set of articles referenced by the citing paper, and asked whether the referenced paper falls in the largest connected component of that network (Fig. 2*C*). Falling outside that largest component might convey information that the referenced paper is less related to the main body of relevant work.

The third and final category of information we included was the content similarity of the citing and referenced articles. Recent advances in large language models have advanced the comparison of the similarity of scientific documents. A recently developed deep learning model, SPECTER (22), has also integrated network-aware contextual information during training to teach the model whether the documents are also likely to have a direct citation linking them in addition to being semantically similar. This pretrained deep learning model, when used for inference, jointly provides information about the semantic and citation network similarity. It is based on the transformer architecture used by many modern large language models, but also encodes information about the likelihood that two articles are linked by citations as well as text similarity. We generated features using SPECTER that encode cosine similarity information for the citing—and referenced article pairs, as well as information about the similarity of the citing article to the rest of its reference list (mean similarity and SD), and the referenced article to the other papers in the reference list (mean and SD). Finally, to capture information about the cohesiveness of the overall reference list, we generated features encoding the similarity of all of the references to one another (mean and SD). Feature importance scores, selection methods, and analysis are shown in the *SI Appendix*.

### Model Training and Validation.

Collectively, these features comprehensively encode information about the citing and referenced articles, their relationships to one another, and the overall context of the other articles found in the reference list. These features have the advantage of being available at the time of publication and not reliant on additional information only disclosed after publication. We assembled balanced training and testing datasets where each instance represented information about the content network regarding a single citing/referenced article pair. For the outcome measure, we applied a binary classification indicating whether this was a citation that was added in peer review (positive, late-stage) or retained from the original preprint (negative, early-stage). Some features that were initially promising were empirically tested and dropped from the final modeling process (*SI Appendix*, Supplemental Materials). We tested random forests, logistic regression models, support vector machines, and extreme gradient boosting (XGBoost). XGBoost (39) was selected for the remainder of the project because it had the highest predictive accuracy of these algorithms.

**Fig. 3.**
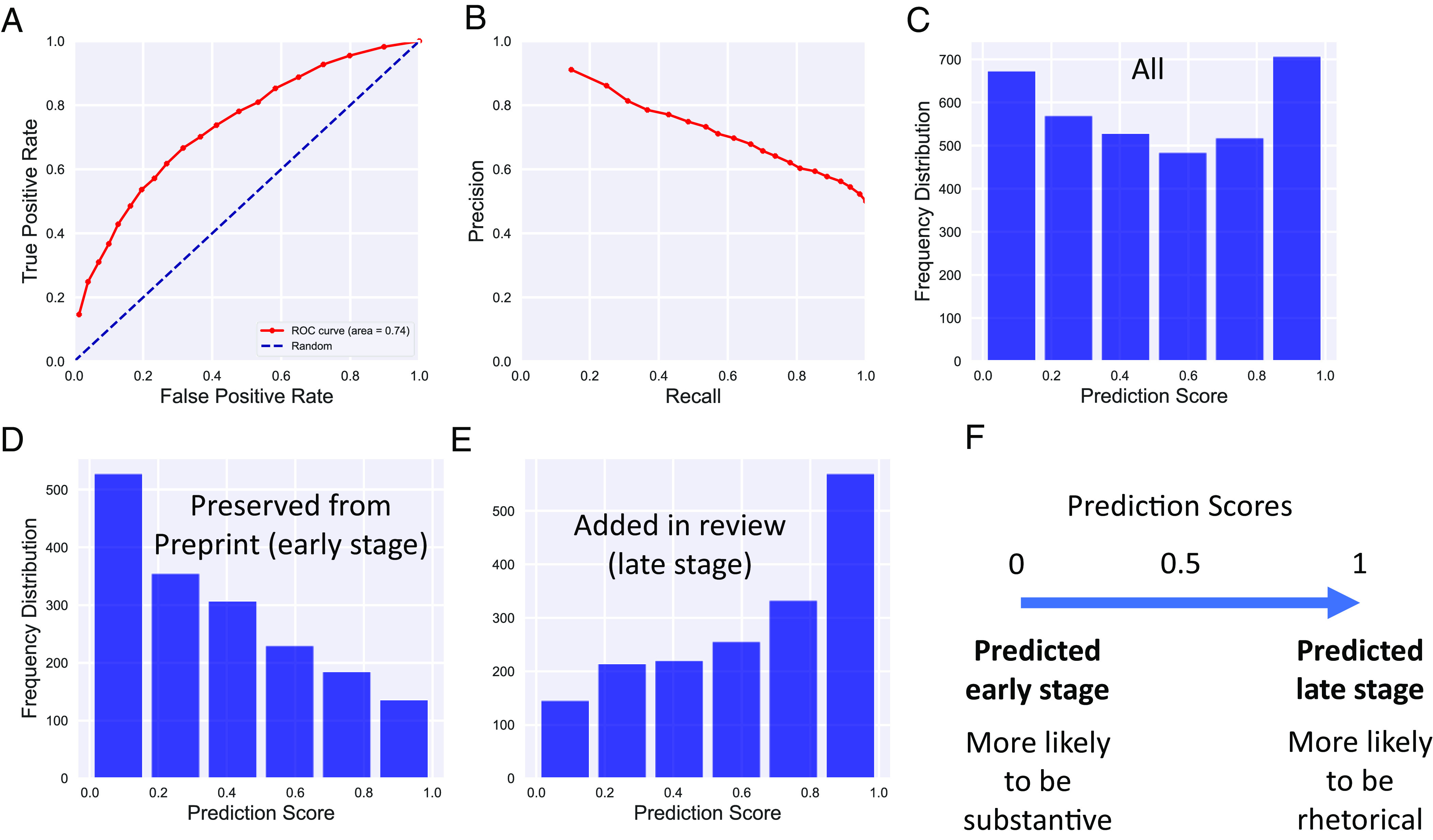
Model accuracy. (*A* and *B*) Precision/recall graph and ROC curve of prediction scores on out-of-sample test data. (*C*) Distribution of prediction scores on a balanced set of out-of-sample positives and negatives (mean 0.5). (*D*) Distribution of prediction scores for out-of-sample positives. (*E*) Distribution of prediction scores for out-of-sample negatives. Low scores can be thought of as a prediction that a citation is early stage, and high scores as late stage. (*F*) Low scores predict that a citation was early stage, and these may be more likely to be substantive (Fig. 1). High scores predict that a citation was late stage, and these might be more likely to be rhetorical.

Overall, the model achieved an F1 accuracy score of 0.7 (Fig. 3) on the test dataset, compared to a chance rate of 0.5. This corresponds to a reduction in uncertainty by approximately 40%. The distribution of prediction scores on the test dataset after training is balanced, but bimodal rather than uniform, weighted toward the ends (Fig. 3*C*).

This level of accuracy is notably lower than studies that seek to identify substantive references and that take advantage of the full citation context. To conceptualize why this is the case, it may be helpful to consider the quantity and quality of information available to the respective types of model. A predictor, whether human or algorithmic, might be confident raising attention to a reference as possibly substantive if it knew that the referenced paper was cited five times in the citing paper, three time in the Introduction section, once in the Methods, and once in the Results, and especially if there was a strong sematic overlap between the citing sentence and the referenced abstract. Conversely, a predictor might overlook a reference that was only cited once, in the Discussion section, with poor semantic overlap between the citing sentence and the referenced article’s abstract. Unfortunately, that information is only available for the small minority of open-access articles. Instead, it may be helpful to consider a task more similar to that described here: A highly cited animal-focused article cites a cellular biology paper with few other citations and with a modest degree of overlap between the respective abstracts. Without knowledge about where and how the referenced paper was cited, and including semantic information related to the citation, the prediction problem becomes much harder. The latter task is what is being modeled here, not to advance the theoretical upper bound on accuracy given all possible information, but to discern the predictive power of the limited features that are universally available at the present time. This necessarily limits the predictive power of our modeling approach, but has the advantage of extending predictive analytics to any biomedical citation rather than only the small minority with free full text available.

We next asked how the predictions of late-stage references related to classes of citations that are a priori more likely to be rhetorical in nature. First, we turned to citations that were present in the preprint version of a paper but were excluded in the published version, reasoning that references that were dropped during review were less likely to be substantive in nature and therefore more likely to be rhetorical. One caution about this dataset is that there is more than one reason that the citation could be dropped. First, an author may have deemed the citation unimportant. In this case, the interpretation is relatively straightforward.

**Fig. 4.**
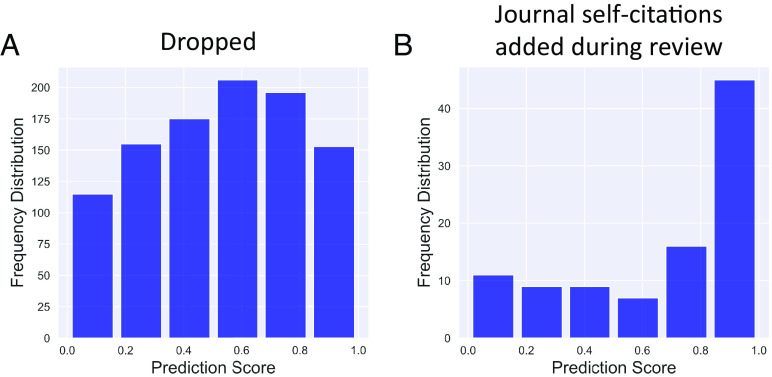
Prediction scores are higher for citations that a priori less likely to be substantive. (*A*) Distribution of prediction scores for preprint references that were dropped in peer review and no longer appeared in the published version. (*B*) Distribution of prediction scores for possibly coercive citations, those that were added in peer review and cite the same journal that the paper was submitted to.

However, a second reason the citation might not appear in the published version has to do with an idiosyncrasy of the data processing pipelines of preprints vs. publishers. bioRxiv includes supplemental references in its online structured citation data. Many publishers do not, and so the citations may not have been dropped at all, but rather were in the supplement the whole time. Nevertheless, because both mechanisms can lead to the apparent removal of the citation, it stands to reason that fewer substantive citations exist in the corpus of dropped citations than those that persisted through review. Thus, despite the fact that these citations are indeed early-stage, if the model is learning about substantive vs. rhetorical citations, these early stage out-of-sample citations should have higher than average (e.g., late-stage) prediction scores if the model is learning features correlated with rhetorical references. If the model only learned about early- vs. late-stage citations in the temporal sense and failed to generalize to the substantiveness of a reference, then these references should score low on the late-stage prediction scores because the ground truth is that they were added early in the study. Fig. 4*A* shows that this is not the case—dropped (and therefore less likely to have been substantive) citations received late-stage prediction scores associated with rhetorical citations despite in fact being early stage. (*P* = 0.0011, Wilcoxon rank sum test). This finding supports the hypothesis that the model may have learned about the substantiveness of a reference by training on references that were added at different stages.

We next turned to a class of citations that has gathered much scrutiny because of their potential for gaming of citation statistics. These are citations that were added in peer review and cite earlier papers from the same journal. These citations are highly suspect; editors insisting on the addition of such citations, in order to game journal-level citation metrics like the journal impact factor, before acceptance have been termed “coercive citations” (11). Note that authors need not wait for editorial feedback before adding such rhetorical citations. They may instead anticipate more favorable editorial decision-making if the citations are added, and preemptively add these (11). Thus, if the model’s scores correlate with the substantive vs. rhetorical nature of a reference, this class of references should also receive high late-stage prediction scores. It should be noted that the model did not have access to information about whether a reference was added during review. These putative rhetorical coercive citations received the highest late-stage scores of any class of citation we examined (*P* < 0.001, Wilcoxon rank sum test and Fig. 4*B*), even the out-of-sample positives. This indicates an association between late-stage citations and two classes that we have a priori reason to be enriched in rhetorical citations.

### External Validation on Classes of Substantive Citations.

Many machine learning models, in addition to learning patterns in the training data to identify the outcome measure that they were assigned, also learn enough about the underlying data structures to apply that knowledge to answer other related research questions. Since the results in Fig. 4 show that the trained model has learned to associate late stage (e.g., high) prediction scores with putative rhetorical citations, we turned our attention to the question of whether it learned about substantive citations as well. We identified five classes of such citations as tests of the trained model’s external validity.

**Fig. 5.**
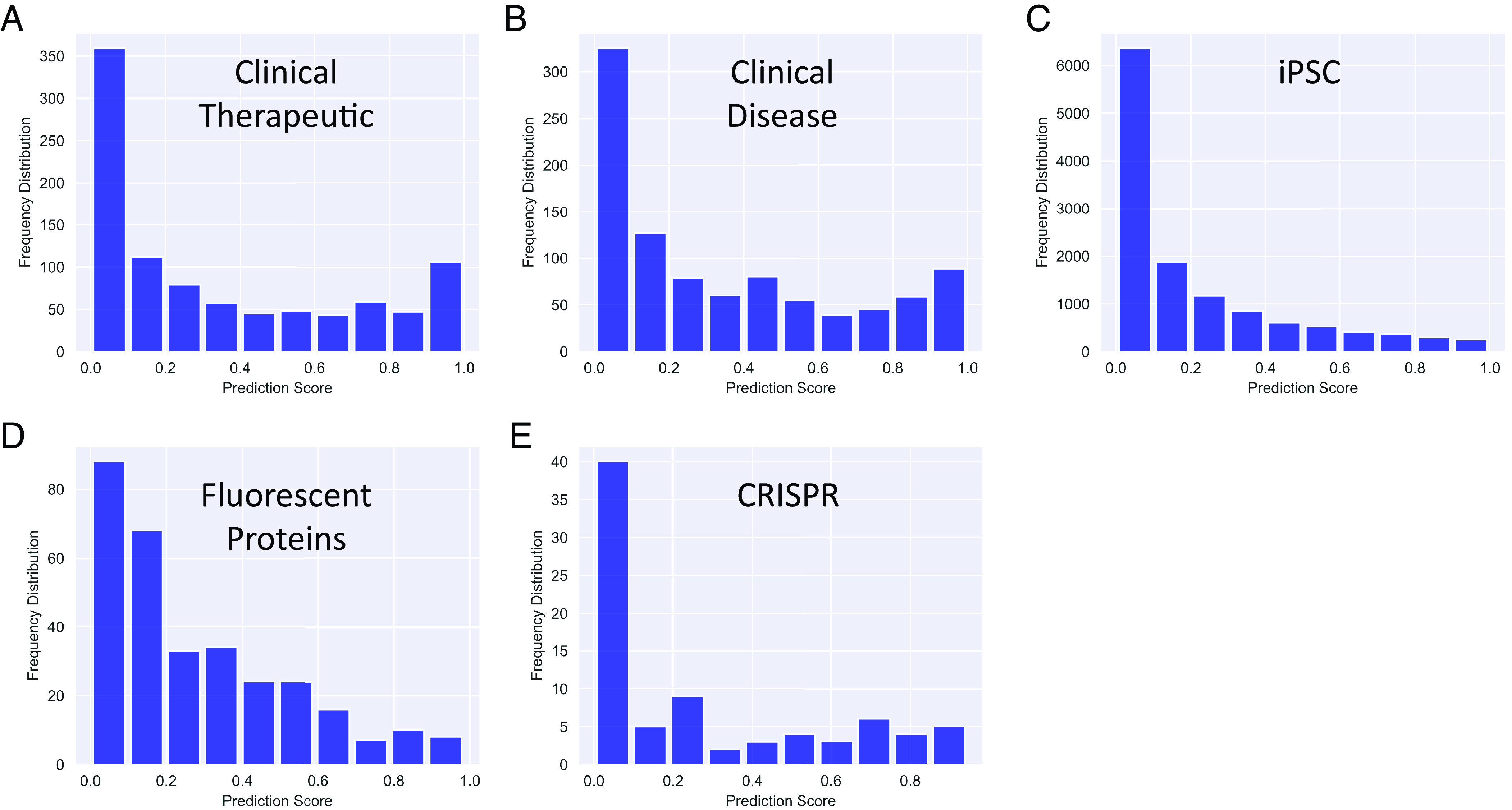
Prediction scores are lower for citations that a priori are known to be substantive. (*A*) Prediction scores for citations from higher phase clinical trials to preceding, lower stage clinical trials matched by the drug therapeutic (mean 0.34). (*B*) Prediction scores for citations from higher phase clinical trials to preceding, lower stage clinical trials matched by the disease being treated (mean 0.34). (*C*) Prediction scores for citations from iPSC papers to the paper describing the seminal iPSC methodology (mean 0.21). (*D*) Prediction scores for citations from papers using fluorescent proteins to the paper describing novel fluorescent proteins for use by the research community (mean 0.28). (*E*) Prediction scores for citations from CRISPR papers to the paper describing the seminal CRISPR gene editing technology (mean 0.27).

First, we relied on the highly structured regulatory framework surrounding drug development to classify substantive citations. In the United States as in many other countries, this framework constrains the generation of clinical knowledge and enforces order to knowledge transfer. Clinical trials proceed in phases that test first for safety and then for efficacy, progressing to larger trials that build off the preceding trials. Therefore, to comply with clinical regulation, later-phase clinical trials for a given drug must draw on previous, lower phase clinical trials assessing that same drug for treating the same disease. These citations from later phase clinical trials to earlier phase clinical trials for the same drug therefore are among the most likely citations to represent substantive knowledge transfer. Our model was trained to identify late-stage citations with high scores. If the model has learned that these classes of substantive references are unlike late-stage references, the prediction scores for this opposite class of citations should have *low* values (e.g., resemble early stage references more closely). Fig. 5*A* shows that this is the case (*P* < 0.001, Wilcoxon rank sum test). Thus, early-stage (low) prediction scores seem to be associated with this class of known substantive knowledge transfer.

We next asked whether the model’s external validity extended to clinical trials studying the same disease rather than the same drug. While perhaps less tightly ordered around the regulatory framework governing drug development, we reasoned that this class of citations, when they occur, are likely to be enriched in substantive citations because of their shared goal of treating the same disease. This is especially likely because some clinical trials are structured to compare novel treatments with the established standard of care, where the regulatory framework encourages knowledge flow within the same disease topic but across different drugs. Here, too, the model gave low prediction scores (Fig. 5*B*), consistent with a successful association (*P* < 0.001, Wilcoxon rank sum test).

We next turned our attention to fundamental research with a study that rapidly emerged into its own research topic. Rather than drawing upon the US regulatory framework, we next drew upon acknowledged contributions to the scientific literature by Nobel Prize-winning research. In the mid-2,000 s, human embryonic stem cell (hESC) research was a novel and promising approach to modeling human biology, contributing to therapeutic development, and being tested as a possible therapeutic agent. At that time, the number of hESC papers in PubMed was growing exponentially. However, there was unmet demand from the scientific community and the public for sources of pluripotent stem cells that carried fewer ethical concerns about abortion and might be subject to less administrative oversight and restriction.

In 2006, the Yamanaka lab produced induced pluripotent stem cells (iPSCs) from adult, rather than embryonic, cells (40). This discovery was followed shortly thereafter in 2007 by production of human iPSCs by the Yamanaka and Thompson labs (41). iPSC research rapidly spread through the cell biology community, and by the mid-2010 s eclipsed hESC research (which had started to decline) and is still growing rapidly. Citations from the later iPSC papers that used or improved upon the original study’s methodology are substantive in nature because the later work would not have been possible without the prior groundbreaking, Nobel Prize-winning work. We identified iPSC papers that cited the original iPSC article (40), and asked whether these, too, received low prediction scores consistent with being an early-stage reference. Fig. 5*C* shows that this is the case (*P* < 0.001, Wilcoxon rank sum test). Thus, this model associates early-stage prediction scores with those that are known a priori to have contributed to the genesis of a new research topic.

Studies that spawn a research topic are rare, and we asked whether enabling technologies that spread rapidly into other established topics are also identified. The development of fluorescent proteins fits this description, although earlier works describing the Green Fluorescent Protein might be considered to have emerged into its own scientific topic. This technology enabled the tagging of individual proteins within the cell, so that their subcellular distribution could be quantified with a genetic approach. For that reason, we examined subsequent fluorescent protein development, when fluorescent protein research had already emerged as a topic of scientific inquiry, but new fluorescent proteins with unique spectral properties represented an enabling technology for other, already established fields of science (42).

In order to identify suitable citations, we turned to the *Methods* section of articles with free full-text in PubMed Central. This is because, unlike the iPSC topic, we sought papers that were not necessarily studying fluorescent proteins per se, but rather were more likely to use them as a means to an end. Open science databases such as OpenCitations and Colil have parsed the citation context (43, 44), which is the sentence in which a reference is cited, alongside the section of the article in which the citation is found. We queried references appearing in the *Methods* section of articles in citation contexts containing names of specific fluorescent proteins that were developed after the emergence of fluorescent proteins as a research topic, but that were still widely used and disseminated in other fields. Like the other classes of citation used for external validation, these are expected to receive low prediction scores if the model associates markers of early-stage references with this substantive knowledge flow. Fig. 5*D* shows that these fluorescent protein citations also receive low prediction scores (*P* < 0.001, Wilcoxon rank sum test). Citations to enabling technologies in two contexts, then, can be detected, without respect to whether the cited article launched a new topic of scientific inquiry.

Finally, we asked whether citations to innovations with particularly high commercial potential are also identified. This is not to say that the commercial potential of iPSCs and fluorescent proteins were not high, but some technologies are known a priori to have especially high commercial potential. Broadly effective genome editing technology is one of these. In 2013, Doudna and Charpentier described a novel gene editing approach using the CRISPR/Cas9 system (45). Subsequently, patent rights for its application were sharply disputed (46). To test whether subsequent citations from papers employing this technique are identified by our model, we again extracted citation contexts from citing papers and included citations to the CRISPR/Cas9 article that appeared in the *Methods* section. This final class of citations that are thought to be substantive in nature also were associated with early-stage prediction scores from the model (*P* < 0.001, Wilcoxon rank sum test and Fig. 5*E*). Thus, the trained machine learning model seems to have identified not only that citations with late-stage prediction scores are less likely to be substantive citations, but also gives low prediction scores (i.e., early-stage prediction values) for known substantive citations (Fig. 3*F*).

### US Federal Support of Fundamental Knowledge Cited by Clinical Studies.

**Fig. 6.**
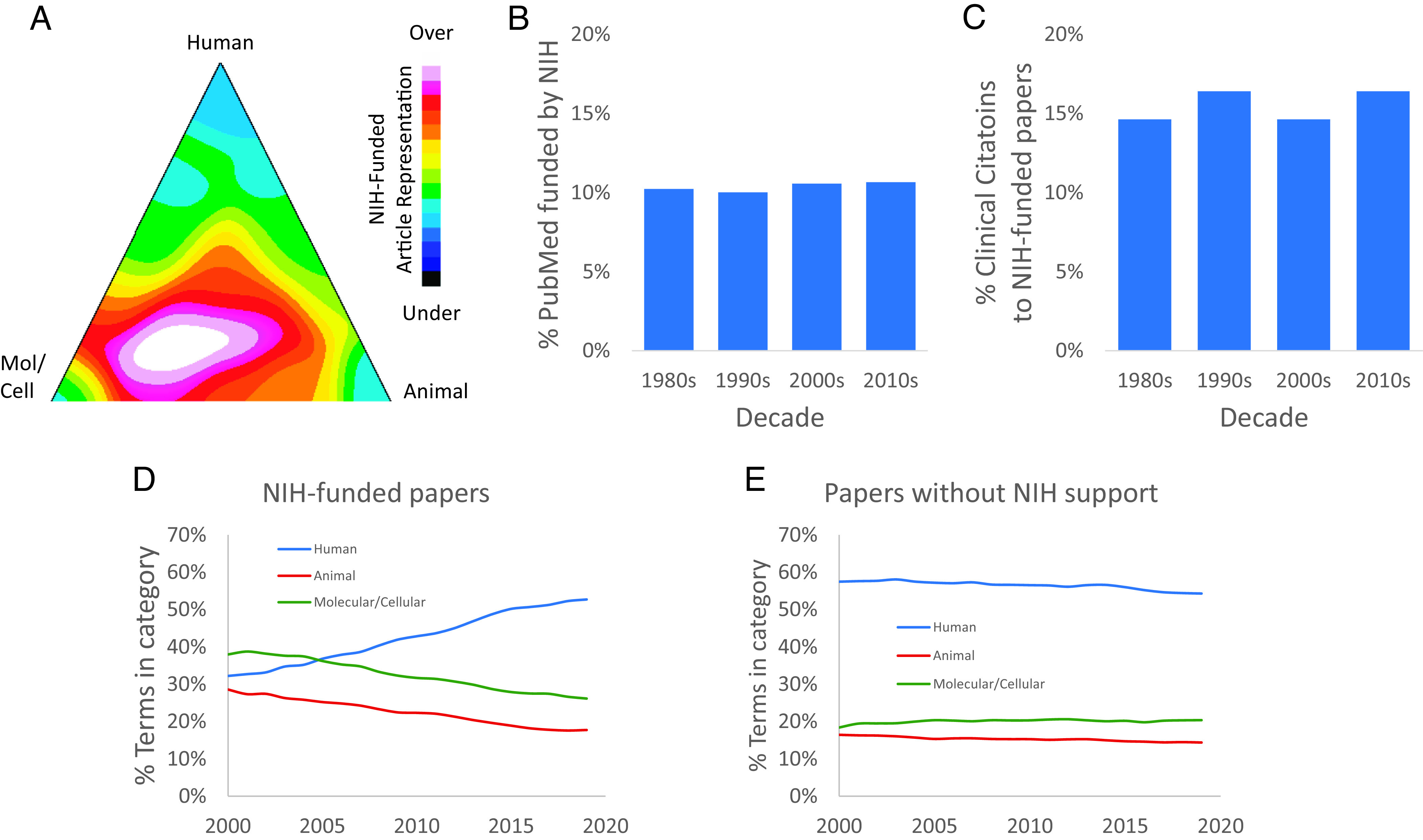
US federal support for human, animal, molecular/cellular biology research. (*A*) Density graph of overrepresentation and under-representation of NIH-funded articles in different domains of the Triangle of Biomedicine. NIH-supported papers are overrepresented in molecular/cellular biology and animal research, and comparatively underrepresented in human research. (*B*) NIH-funded articles comprise ~10% of the biomedical literature. (*C*) NIH-funded articles typically receive 15 to 16% of citations from clinical research articles. (*D*) Shifts of the domains of research funded by NIH over time. (*E*) The non-NIH-funded literature has a strong and stable human focus.

One pressing question in biomedicine is how to more effectively facilitate the generation of knowledge that drives later clinical discovery. However, before applying our model to address this question, it is first necessary to understand the overall dynamics of bench-to-bedside translation in recent years. We focused on the distinction between research funded by the United States federal government vs. research that was not. This is because science funding is a major policy lever easily accessible to lawmakers for advancing science, and the value of government-funded science is under constant scrutiny. Using the human, animal, and molecular/cellular biology classification initially developed by Weber (31) can help to identify the domain of science for each paper. Human publications are conceptually the closest to clinical research, while animal and especially molecular and cellular biology research are conceptually farther. These can be visualized on the Triangle of Biomedicine (3, 31), which visualizes articles in a translational space. Human research articles characterize the majority of biomedical studies. By contrast, NIH-funded research has a more basic research focus (Fig. 6*A*), although human-focused research is an increasingly large part of the NIH portfolio (Fig. 6).

It stands to reason that the more basic-research favored for federal funding might be less-well cited by clinical articles, due to the larger conceptual distance to applied clinical research. Extending this line of reasoning, it may be that those clinical citations that exist might be less likely to represent substantive knowledge transfer because of the large conceptual gap between basic and applied clinical research. Over the period we studied, NIH-funded scientific papers represented 10.6% of biomedical publications (Fig. 6*B*). We therefore asked whether US federally funded research articles represent more or fewer than ~10% of clinical citations as a first test of the representation of US federally funded biomedical research in clinical citations. NIH identifies clinical citations as citations from clinical observational studies, trials, or guidelines in its iCite web service (3). NIH-funded articles comprise 15.7% of papers cited by clinical articles (Fig. 6). Despite the more basic research focus, NIH-funded articles receive nearly 50% more clinical citations than might be expected based on their fraction of the literature. However, because of the limitations of citation analysis, this result does not necessarily reveal the overrepresentation or underrepresentation of *substantive* knowledge flow from federally supported to clinical articles.

### Estimated Substantive Citation in Government-Supported Bench-to-Bedside Translation.

**Fig. 7.**
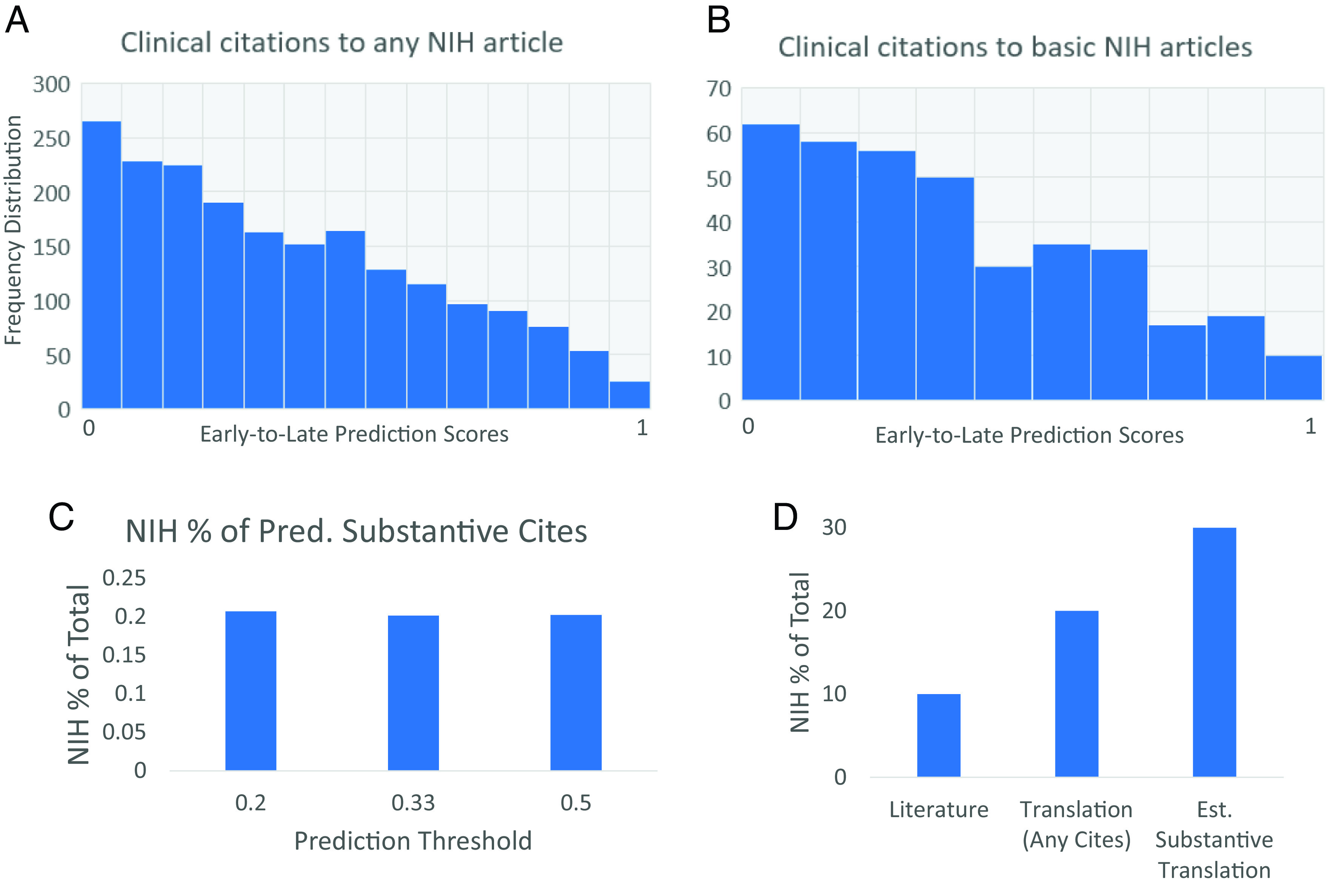
Government support overrepresented in early stage and thereby likely substantive knowledge flow from basic to clinical research. (*A*) Distribution of late-stage scores for clinical citations to any NIH-funded articles. (*B*) Distribution of late-stage scores for clinical citations to basic research papers funded by NIH (referenced papers have Human scores below 0.5). (*C*) NIH-funded papers receive 20% of citations with prediction scores below threshold, regardless of the threshold chosen. (*D*) NIH-funded papers are overrepresented in clinical-to-basic citations [20%, no filtering on prediction score, “Translation (Any Cites)”]. NIH-funded papers comprise nearly a third of basic research articles that receive clinical citations with a late-stage score below 0.2 (most likely to be substantive, early-stage knowledge transfer).

To address questions about substantive clinical citations, we first had to operationalize this concept for the purposes of this study. In our external validation studies involving citations that we have reason a priori to believe represent substantive knowledge flow, like iPSC and progressive phases of clinical trials involving the same drug, these generally scored below 0.5 on predictions from our model (e.g., were predicted to be early stage and therefore more likely to represent substantive knowledge transfer). Clinical citations to NIH-funded articles had a similar distribution of prediction scores (Fig. 7*A*). We asked if this pattern changed when examining clinical citations to *basic* NIH research, given the greater conceptual distance between basic and clinical research. We observed that these clinical citations also had a low distribution of late-stage prediction scores (*P* < 0.001, Wilcoxon rank sum test Fig. 7*B*), and also resembled the external validation datasets that are a priori likely substantive citations.

We acknowledge that lower precision but higher coverage models like this are disadvantaged in accuracy compared to those relying on citation contexts. However, models using exclusively open data represent a substantial improvement in coverage over more precise predictive methods that rely on full text that is only available for ~20% of articles. Our findings indicate there are untapped data sources that can help to fill in the gaps overlooked by the high-accuracy but low-coverage approaches that depend on the availability of free full text.

Based on the model’s prediction scores and the external validation studies shown in Fig. 5, we reasoned that citations with early-stage prediction scores are likely to be more concentrated with substantive citations. To be clear, we do not assert that each individual citation with an early-stage score (i.e. low) is a substantive citation, merely that citations with early-stage prediction scores are more likely to be concentrated with substantive citations relative to those receiving late-stage prediction scores based on our analysis. Using early-stage prediction scores (i.e., those below 0.5) to enrich a pool of substantive citations from clinical articles, we found that NIH-funded publications comprised 20% of such citations, twice as high as the representation of NIH-funded publications in the scientific literature (Fig. 7). Limiting to the predicted early-stage clinical citations thus reveals that, compared to the 15% of clinical citations received by NIH papers overall (Fig. 6), US federally funded studies are overrepresented among those citations most likely to represent early (and thereby associated with substantive) knowledge transfer. Furthermore, this estimate was invariant to the particular threshold used. NIH-funded publications received 20% of the total predicted early-stage clinical citations using more stringent thresholds of 0.33 and 0.2 as well (Fig. 7*C*), the latter of which was the most stringent and the most concentrated in early-stage citations from our external validation (Fig. 5), and therefore used in the next analysis. Thus, independent of the particular threshold used, federally funded biomedical research is overrepresented twofold among clinical citations estimated as associated with early-stage and therefore more-likely substantive knowledge transfer by our model and analysis.

We next asked whether this finding extended to clinical citations to *basic* research articles rather than NIH-funded articles overall. This is because, although less human-oriented than the literature as a whole, the NIH portfolio is increasingly focused on human-focused (3, 31) research articles (Fig. 6), as the share of human terms has nearly doubled over the time frame studied (Fig. 6*D*). Previous work has identified research that is less than 50% human focus as enriched in basic research articles (3, 31). Using that definition, we found that NIH-funded research articles comprised 30% of estimated early-stage citations from clinical to basic research articles (Fig. 7). NIH-funded work comprises only 10% of the literature. It represents an estimated 30% of the basic research literature predicted to be early-stage, and therefore associated with substantive knowledge transfer, into clinical research (*P* < 0.001, Chi-squared test for probabilities).

## Discussion

An important applied goal of biomedical research is to generate knowledge that can advance human health. US federal science funding is among the most prominent policy levers for stimulating basic research knowledge that could inform new therapeutic development. Citations represent knowledge flow from the referenced article to the citing one, and can be used to trace the movement of basic research ideas into such downstream applied research. Recently we developed a large-scale database of citations from the clinical literature to the rest of biomedical science, which for the first time can be used to address these kinds of questions (2, 3, 32). However, the presence of a citation does not reveal how that knowledge was put to use. Some references are exclusively rhetorical in nature, and may not have been meaningfully built upon in the citing study.

Our results demonstrate that it is possible to use information about local network structure (Fig. 2 and *Methods*), deep learning large language models of semantic representations of text similarity (*Methods*), and article metadata (2, 3, 28, 34, 47) to discern a signal of citation substantiveness from the noisiness of citation dynamics. We took advantage of knowledge from preprints and their published versions to identify a class of citation that is, a priori, less likely to have been substantively used: those added during peer review, after the main body of work has been completed (7). We then trained a model to predict when a reference is likely to have been added at a late stage of a work and thereby less likely to have contributed substantively. We found that citations that are known historically to have been substantive in nature are more likely to be predicted as early-stage references, and conversely, those that are a priori more likely to be rhetorical in nature are likely to be scored as late-stage, whether they were in fact late stage or not. Finally, we apply this model and find that federally funded publications are vastly overrepresented in the population of basic-to-clinical citations with early-stage prediction scores. This population of citations is liklier to represent substantive knowledge transfer in a bench-to-bedside translation context, raising the possibility that federal support for biomedical research is an effective tool for stimulating this process.

This study has important limitations. First, because of the scope of this work and the data utilized (2, 28, 32), it is limited to biomedical research fields. It is possible that the model efficacy at identifying substantive citations would change if expanded to other fields. Second, since preprint data are relatively recent in biomedicine, it may be the case that this modeling approach is most effective at making predictions about recent citations, rather than historical ones from decades past. This is especially salient because the later publications analyzed contain COVID-19 publications. It is not known if citation or semantic relationships shifted during the pandemic. Third, there may be a mixture of substantive and rhetorical references in both early and late stages (although at different frequencies), which makes this outcome a noisy measure. It is not known whether the network structures and semantic representations used for feature generation drift over the scope of decades. However, it is our anticipation that further research into identifying substantive citations without full text will increase the accuracy and coverage of information about substantive vs. rhetorical knowledge transfer. Finally, it is unclear what the relationship is between citation to government-funded articles and early-vs. late citations represents. We anticipate future work will identify effective methods to combine the approaches reported here with the rich information for citations where contexts are fully available.

Evidence from the literature supports the interpretation that citations added during review are less likely to be substantive, leaving early stage references with a proportionately higher level of these. This is because biomedical articles do not change much during the review process, leaving little scope for this stage of biomedical science communication to effect change (48–52). Over 80% of bioRxiv authors self-report posting preprints before receiving reviews (50). Studies comparing preprints with their peer-reviewed counterparts have analyzed the credibility of preprints as well as the effectiveness of peer-review. One (53) manually compared the quality of reporting of both independent and paired preprint and peer-reviewed samples from bioRxiv and PubMed. Quality of reporting in preprints and peer-reviewed articles is within a comparable range. Brierley et al. analyzed the preprints recording the early phase of the COVID-19 pandemic and their subsequently peer-reviewed versions (50). The number of figures, panels, and tables showed little difference on average, suggesting that very few new experiments or analysis were conducted during the peer-review. The semantic analysis of the title, abstract, and body of the preprints in arXiv and bioRxiv found little notable difference with their peer-reviewed versions (48). Comparison of preprints and their peer-reviewed versions on COVID-related research also found little difference in the peer-reviewed results (48, 53, 54). Analysis of the primary data constituting the evidence base suggests that estimate values in published papers are nearly identical to those in preprint versions (55, 56). Few measurable differences in peer review evaluations of article quality exist between preprints that are published vs. those that remain unpublished (55). Studies that examined non-COVID preprint-publication pairs drew similar conclusions (49, 57–60). These studies support the use of preprints, as one important component of the scientific literature, in downstream research development and decision-making.

The methods explored here should not be viewed as an alternative for citation context-based approaches for identifying substantive citations (9, 18–22). These two approaches, although aligned in their goals, utilize nonoverlapping information, and the insights from each type of model can be combined to further reduce uncertainty about substantive knowledge flow. Instead, the approaches used here can be used to address the large coverage gap in open access to free full text, and dramatically expand the scope of citations that can be examined from the context of importance. We show that it is possible to generate predictions about substantive citations even without access to full text citation context.

## Methods

### Raw Data & Code Availability.

Preprints that have been published were identified through the Europe PMC application programming interface, which matches the DOI of the preprint and published versions of a paper. DOIs corresponding to bioRxiv preprints were identified and their full text, which includes a structured reference list, were downloaded in October 2020 (55). We matched 38,378 bioRxiv preprints to published papers indexed in PubMed, spanning the time period from 2013 to 2020. Although some papers overlapped with the COVID-19 pandemic, most were published prior to that event. We included all areas of science indexed in bioRxiv, but this may not completely overlap in terms of topic with PubMed as a whole; in particular, in earlier years, bioinformatics may be overrepresented since this community adopted preprinting earlier than others. It should be noted that it is possible that more recent preprint-publication linkage may not yet have been indexed in Europe PMC due to possible reporting or data processing delays. For this reason, more recent preprint-publication links may be underrepresented in our sample. References were matched with the National Library of Medicine’s Hydra citation resolution service (4). We used the NIH Open Citation Collection to identify references from the final, published version of a manuscript (2, 28). We used publication-grant linkages from NIH ExPORTER to identify federally supported biomedical research articles (61).

Many of the derived features described below used data from the iCite database (e.g., Human, Animal, Molecular Biology scores (3), or the RCR (33, 34) in its linear or percentiled form) (2, 3, 28, 34, 47). These are available at the Figshare data repository (47). Raw text for similarity comparisons are available at PubMed (62). Source code for the SPECTER and XGBoost libraries used in this study are available online (22, 63, 64).

See *SI Appendix* for additional details about feature descriptions, machine learning models and accuracy testing, and external validation analysis.

## Supplementary Material

Appendix 01 (PDF)

## Data Availability

Bibliometric metadata have been deposited in Figshare (10.35092/yhjc.c.4586573) (64).
