# Supplementary material for: Predicting substantive biomedical citations without full text
Source: Proc Natl Acad Sci U S A. 2023 Jul 18;120(30):e2213697120. doi: 10.1073/pnas.2213697120 (PMC10372685; doi:10.1073/pnas.2213697120)
Supplement: Supplementary file 1 — Appendix 01 (PDF) [file pnas.2213697120.sapp.pdf]

Supplemental materials for: Predicting substantive biomedical citations without full text

Supplemental Methods

Feature descriptions

- `specter_cosine_sim`: SPECTER cosine similarity using the title and abstract text of the citing paper and that of the referenced paper
- `pub_ref_reflist_sd`: Standard deviation of the pairwise SPECTER cosine similarities using the title and abstract text of the referenced paper and that of all other papers in the citing article's reference list
- `pub_ref_reflist_mean`: Mean of the pairwise SPECTER cosine similarities using the title and abstract text of the referenced paper and that of all other papers in the citing article's reference list
- `pmid_reflist_sd`: Standard deviation of the pairwise SPECTER cosine similarities using the title and abstract text of the citing paper and that of all other papers in the citing article's reference list
- `pmid_reflist_mean`: Mean of the pairwise SPECTER cosine similarities using the title and abstract text of the referenced paper and that of all other papers in the citing article's reference list
- `reflist_reflist_sd`: Standard deviation of the pairwise SPECTER cosine similarities using the title and abstract text of all papers in the citing article's reference list to one another
- `reflist_reflist_mean`: Mean of the pairwise SPECTER cosine similarities using the title and abstract text of all papers in the citing article's reference list to one another
- `ref_year`: Publication year of the referenced paper
- `ref_rcr`: Relative Citation Ratio of the referenced paper
- `pub_rcr`: Relative Citation Ratio of the citing paper
- `cocited_by_ref`: Count of the number of other papers in the citing paper's reference list that also cited the referenced paper
- `pub_pctile`: Percentile of the citing paper's Relative Citation Ratio
- `ref_mc`: Molecular/Cellular Biology score of the referenced paper. This is the average of relevant Medical Subject Heading terms attached to this paper that fall into the Molecular/Cellular category
- `pub_mc`: Molecular/Cellular Biology score of the citing paper. This is the average of relevant Medical Subject Heading terms attached to this paper that fall into the Molecular/Cellular category
- `ref_a`: Animal score of the referenced paper. This is the average of relevant Medical Subject Heading terms attached to this paper that fall into the Animal category
- `pub_a`: Animal score of the citing paper. This is the average of relevant Medical Subject Heading terms attached to this paper that fall into the Animal category

- `pub_year`: Publication year of the citing paper
- `ref_pctile`: Percentile of the referenced paper's Relative Citation Ratio
- `ref_h`: Human score of the referenced paper. This is the average of relevant Medical Subject Heading terms attached to this paper that fall into the Human category
- `pub_h`: Human score of the citing paper. This is the average of relevant Medical Subject Heading terms attached to this paper that fall into the Human category
- `in_lcc`: Binary flag for whether the referenced paper falls into the largest connected component of the local citation network of the papers in the citing article's reference list
- `direct_and_cocitation`: Binary flag for whether the direct citation between the citing and referenced paper is also a co-citations; in other words, has another article been published after the citing paper that referenced both the citing and cited paper?
- `ref_is_research`: Binary flag for whether the referenced paper is a primary research article
- `same_journal`: Binary flag for whether the citing and cited papers are both published in the same journal
- `pub_is_research`: Binary flag for whether the referenced paper is a primary research article

Machine learning & accuracy testing

Our outcome measure for prediction was a binary flag indicating whether a given reference had been added in peer review (True) or was found in the original preprint and carried over to the published version (False). Training data contained 440,000 instances of balanced positive and negative data, and accuracy statistics were calculated from a smaller holdout test set. XGBoost was used for the final model (1), although we also tested random forests, support vector machines, and logistic regression (each of which had poorer performance). The model achieved an F1 score of 0.7. Training and testing using the complete balanced dataset with 10-fold cross-validation yielded similar results. Our final test set comprised of 3482 citation linkages that were out-of-sample for training the model.

References that were found in the preprint version but not in the published version were omitted because of a difference in how such references are indexed. bioRxiv and medRxiv host both the primary reference list and references found only in the supplemental material, while many publishers do not deposit references from supplemental material into structured citation indices. It cannot be easily distinguished whether a reference is found in the preprint but not the published version due to being genuinely dropped, or whether it was part of the supplemental data and not covered by publishers. We therefore omitted such references from training data.

External validation & analysis

To identify earlier stage clinical trials cited by later stage clinical trials for the same drug, we used PubChem to match drugs and PubMed Publication Type terms to identify which Phase (I-IV) each citing/referenced paper was (2). To identify those trials studying the same disease, we matched based on the Disease Medical Subject Heading terms available in PubMed. To test the role of federal support on estimated substantive translation on clinical citations, we randomly sampled 10,000 citations from clinical papers published in 2015-2019 for subsequent subsetting and analysis. Sample sizes for other external validation conditions were: Clinical progression of the same drug (955); clinical progression of the same disease (958), iPSC (12693), XFP (312), and CRISPR (81).

Some external validations required examining the location in the citing paper of the citation. Such citation contexts are available from OpenCitations and Colil (3, 4); we used context data from the latter service. Only citations in sections that contained the case-insensitive string “methods” in the header were used for matching citations in the Methods section (e.g. “Methods”, “Materials and Methods”, or “Methods and Results”). Additional citations to the same paper in other sections of a paper did not exclude a reference from consideration as long as it was also cited in the Methods section.

Supplemental Results

Feature importance scores

Feature importance scores are shown in Supplemental Table 1. Out-of-sample positives received a higher prediction score of 0.63 on average, while out-of-sample negatives received an average prediction score of 0.37 ($p < 0.01$, Wilcoxon rank sum test, and Figure 3d-e). This is important because it is possible for a prediction system to have predictive power mainly for identifying positive values, while performing closer to chance at identifying negatives. If that were the case, it would indicate that the model had learned some predictive properties of papers added in peer review, but little else. The low distribution of prediction scores for the out-of-sample negative samples indicates that the model is learning patterns about early-stage citations as well.

Feature	Gain	Cover	Frequency
specter_cosine_sim	0.099	0.137	0.116
pub_ref_reflist_sd	0.098	0.149	0.120
pub_ref_reflist_mean	0.094	0.152	0.115
pmid_reflist_sd	0.081	0.096	0.071
pmid_reflist_mean	0.081	0.098	0.069
reflist_reflist_sd	0.077	0.101	0.069
reflist_reflist_mean	0.076	0.095	0.066
ref_year	0.067	0.019	0.050
ref_rcr	0.062	0.047	0.074
pub_rcr	0.035	0.023	0.032
cocited_by_ref	0.025	0.008	0.020
pub_pctile	0.025	0.018	0.021
ref_mc	0.024	0.005	0.028
pub_mc	0.023	0.005	0.021
ref_a	0.021	0.004	0.024
pub_a	0.019	0.004	0.017
pub_year	0.018	0.003	0.011
ref_pctile	0.018	0.020	0.020
ref_h	0.018	0.005	0.021
pub_h	0.017	0.004	0.016
in_lcc	0.010	0.002	0.006
direct_and_cocitation	0.006	0.002	0.006
ref_is_research	0.005	0.001	0.005
same_journal	0.002	0.001	0.002

pub_is_research	0.001	0.001	0.000

Supplemental Table 1. Feature importance scores from the trained model. See Methods for full feature descriptions. Gain, fractional contribution of features, indicating a more predictive feature. Cover, relative number of observations related to the feature. Frequency, a count of the relative number of usages of a feature within trees.

Feature space analysis

We first began with features extracted from three databases: PubMed, iCite, and Semantic Scholar. This initial model used the features described in the Methods: Feature Descriptions section (which derived data from iCite and PubMed), as well as four additional features derived from Semantic Scholar (Supplemental Table 2). This model, in addition to being more complex, also requires ingesting a third database, so its upstream data processing is also more complex. The four features derived from Semantic Scholar data each received feature importance scores near the bottom the importance ranking (Supplemental Table 3), so we explored creating a model without these (background, methods, results, authors_pmid_ref_overlap). Both models received similar F1 scores: 0.7 for the model including Semantic Scholar data, and 0.7 for the model using only PubMed and iCite data (see Results). A heatmap of the feature correlation matrix is shown in Supplemental Figure 1. Because these two scores were similar, we used the model without Semantic Scholar data to simplify the model and data processing pipeline.

Additional feature	Description
background	Semantic Scholar's estimate, based on their deep learning model of citation contexts, that this citation is a 'background' style citation
methodology	Semantic Scholar's estimate, based on their deep learning model of citation contexts, that this citation is a 'methodology' style citation
result	Semantic Scholar's estimate, based on their deep learning model of citation contexts, that this citation is a 'result' style citation
authors_pmid_ref_overlap	Measure of overlap between the authors' published papers and those referenced in this paper (a measure of self-citation). Uses Semantic Scholar author publication profiles

Supplemental Table 2. Additional features removed from final model.

Feature	Gain	Cover	Frequency
specter_cosine_sim	0.094354	0.132561	0.112403
pub_ref_reflist_sd	0.094085	0.148231	0.117254
pub_ref_reflist_mean	0.090423	0.148164	0.111646
pmid_reflist_sd	0.079526	0.098855	0.071388
pmid_reflist_mean	0.077426	0.09796	0.06851
reflist_reflist_sd	0.076695	0.098471	0.068842
reflist_reflist_mean	0.076608	0.097763	0.066528
ref_year	0.065068	0.019641	0.048819

ref_rcr	0.060807	0.046601	0.072988
pub_rcr	0.034135	0.022887	0.030774
pub_pctile	0.025242	0.017468	0.021312
cocited_by_ref	0.024214	0.007907	0.019668
ref_mc	0.024012	0.004573	0.028233
pub_mc	0.022668	0.004451	0.020438
ref_a	0.020082	0.003833	0.023841
ref_pctile	0.018071	0.018312	0.02027
pub_year	0.017811	0.003961	0.010814
pub_a	0.017683	0.003971	0.016499
pub_h	0.016961	0.003959	0.015513
ref_h	0.016905	0.004209	0.020117
in_lcc	0.00921	0.001785	0.00549
background	0.009112	0.002059	0.006828
authors_pmid_ref_overlap	0.008146	0.005574	0.005312
direct_and_cocitation	0.005883	0.001672	0.005696
methodology	0.005352	0.001125	0.003284
ref_is_research	0.005143	0.001319	0.004551
same_journal	0.001666	0.000801	0.001553
result	0.001614	0.001074	0.00093
pub_is_research	0.001097	0.000816	0.000499

Supplemental Table 3. Feature importance scores of the more complex model.

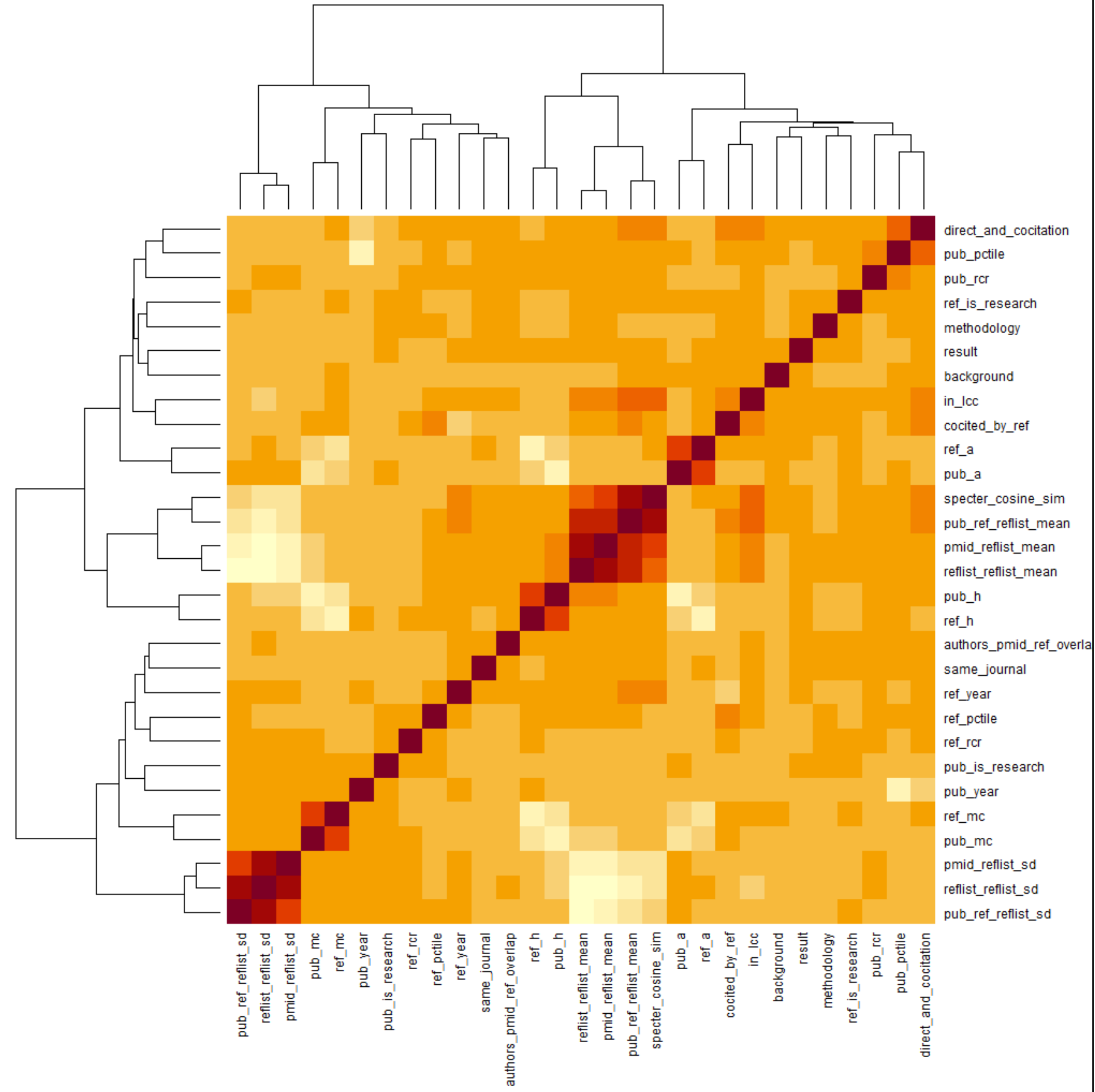

Supplemental Figure 1. Feature correlation matrix displayed as a heatmap, based on the training data.

Feature reduction analysis

To further test whether the model complexity could be reduced without sacrificing accuracy or external validity, we collapsed pub_year and ref_year into a composite variable dyear by subtracting ref_year from pub_year. Based on feature importance scores, we also removed two of the three local network structure features (direct_and_cocite and in_lcc). In addition, we removed the Human, Animal, and Molecular/Cellular scores, and retained only the percentiled RCR scores (pub_pctile and ref_pctile) while omitting the linear RCR scores. Finally, we removed the same_journal and is_research flags. We retained all the SPECTER data because these features received among the highest feature importance scores.

Our reduced feature set generated a model with similar levels of accuracy (F1 = 0.69 double check this). This indicates that raw accuracy was not much affected by the reduced feature space. However, when we checked its external validity on our five examples of known substantive knowledge transfer (clinical drug progression, clinical disease progression, iPSC, XFP, and CRISPR), only four out of five examples showed the expected pattern of low prediction scores. This indicates that some external validity may have been traded off by reducing the feature set in this way. For this reason, we continued our experiments with the main model as described in the main text (full iCite + PubMed features but no Semantic Scholar features).

References

1. Community DDML. XGBoost: eXtreme Gradient Boosting 2016 [Available from: https://github.com/dmlc/xgboost/.
2. Medicine NLo. Download MEDLINE/PubMed Data 2020 [Available from: https://www.nlm.nih.gov/databases/download/pubmed_medline.html.
3. Fujiwara T, Yamamoto Y. Colil: a database and search service for citation contexts in the life sciences domain. J Biomed Semantics. 2015;6:38.
4. Peroni S, Shotton D. OpenCitations, an infrastructure organization for open scholarship. Quantitative Science Studies. 2020;1(1):428-44.
